# The Effect of Drawing in Conventional and Hydrodynamic Dies on Structure and Corrosion Resistance of Hot-Dip Galvanized Zinc Coatings on Medium-Carbon Steel Wire

**DOI:** 10.3390/ma15196728

**Published:** 2022-09-28

**Authors:** Maciej Suliga, Radosław Wartacz, Marek Hawryluk, Joanna Kostrzewa

**Affiliations:** 1 Faculty of Production Engineering and Materials Technology, Czestochowa University of Technology, 19 Armii Krajowej Av., 42-201 Czestochowa, Poland; 2 Independent Researcher, 1b Mykanowska Str., 42-240 Kościelec, Poland; 3 Department of Metal Forming and Metrology, Wroclaw University of Science and Technology, 5 Lukasiewicza Str., 50-371 Wrocław, Poland; 4 Faculty of Technology, The Jacob of Paradies University, 52 Fryderyka Chopina Str., 66-400 Gorzów Wielkopolski, Poland

**Keywords:** wire, hydrodynamic dies, zinc coatings, microstructure, corrosion

## Abstract

The paper presents the impact of the drawing method on the microstructure and corrosion resistance of galvanized steel wires. The microstructural tests confirmed that, in the drawing speed range v = 5–20 m/s, the use of hydrodynamic dies creates more favorable conditions for the deformation of the soft zinc coating on the hard steel core. The increase in friction at the wire/die interface in the conventional method, as compared to the hydrodynamic method, contributed to the decrease in coating thickness and the increase in the diffusion layer, and the higher the drawing speed, the greater the differences between the analyzed drawing methods. In the conventional method, while drawing at high speeds v = 20 m/s, there was a two-way diffusion and complete remodeling of the ζ phase in δ_1_. In the hydrodynamic method, at the speed of 20 m/s, in the analyzed micro-areas, places showing the presence of the ζ phase, partially dispersed in the layer with pure zinc, were observed. A corrosion tests comparison between conventionally and hydrodynamically drawn wires showed an improved behavior of the latter. The greater mass in the surface layer of pure zinc, a substrate for the corrosion product in hydrodynamically drawn wires, reacted, creating insulation from the white corrosion produced. The compressive stresses in the hydrodynamic dies caused by the high pressure of the lubricant on the circumference of the wire closed the microcracks on its surface, which additionally sealed the zinc coating.

## 1. Introduction

The wires and wire products with increased corrosion resistance are used in the production of springs, ropes, nets, and fasteners. The corrosion resistance largely depends on the type of metallic anti-corrosion coating applied to the surface of the steel wire (zinc, brass, etc.) [1,2,3,4,5,6,7,8] and its thickness. The other factor significantly affecting the corrosion resistance of the steel substrate is the method of applying the coatings, which determines the thickness of the anti-corrosive layer (galvanic, immersion, thermodiffusion methods) [9,10,11,12,13,14,15], and the third is the wire drawing technology [16,17,18,19]. The same technical problem occurs in the drawing process, regardless of the type of coating. A wire with a metallic coating is, in a sense, a bimetallic product made of a hard steel core and a thin, soft metallic coating with anti-corrosion properties. The various rheological properties of materials and the friction phenomenon in the area of the die and wire, causing uneven distribution of stresses and strains, disturb the metal flow in the surface layer of the wire. Consequently, the result is a more significant thinning of the coating than it would from the reduction in the cross-section (the material elongates with the successive pull), and it is related to the tearing and crushing of the coating on the wire surface. These phenomena also occur in the case of drawing galvanized wires.

The hot-dip zinc coatings are multilayer coatings made of unalloyed and intermetallic alloy phases of zinc and iron [20,21,22,23,24,25]. The thickness and distribution of these phases in the coating depend on many factors, including steel grade, galvanizing parameters—time, temperature, composition of the zinc bath and galvanizing technology [26,27,28,29,30,31]. In a nutshell, galvanizing consists of pickling and rinsing a wire rod and immersing it in liquid zinc (hot-dip method) or an electrolyte (galvanic method). Depending on the galvanizing method, the thickness of the anti-corrosion coating is from a few to over 50 µm. Then, the material prepared in such a way is pulled, using multistage drawing machines, onto a wire of a specific diameter [32].

The technology of drawing steel wires depends to a large extent on the diameter of the material after galvanizing. Coarse and medium wires are drawn dry with the use of drawing powders, while thin wires are wet drawn in emulsions containing calcium, sodium, water and other chemical additives. The basic parameters which play a key role in wire forming are: die geometry, lubricants, drawing speed and drawing method [33,34,35,36,37,38,39]. Changing the drawing method enables a significant reduction in friction at the wire/die interface. It is assumed that the coefficient of friction when drawing in conventional dies is, on average, about 0.07, while when drawing in hydrodynamic dies, it is possible to obtain from 0.007 to 0.04 as a result of high pressure of lubricant in the drawing dies.

The authors’ research published in paper [40] confirmed the significant influence of the drawing method on wire properties. The results of this work showed that, regardless of the drawing speed, the use of hydrodynamic dies has a positive effect on the drawing process and partially eliminates the negative influence of friction on the flow of the wire surface layer containing the zinc coating. The use of the hydrodynamic dies, as compared to conventional drawing, allows for obtaining a thicker layer of lubricant in the drawing process, which protects the surface of the wire against tearing during its passage through the drawing. Hence, wires drawn in hydrodynamic dies, as compared to wires drawn in conventional dies, are characterized by better plastic and technological properties and lower residual stress. This study is a continuation of these studies, in which the authors explain the influence of multistage drawing in hydrodynamic dies on structural changes of the zinc coating and its corrosion resistance. This is a new issue that has not yet been described in the literature available.

## 2. Materials and Methods

### 2.1. Manufacturing Process of Hot-Dip Galvanized Steel Wires

The test material was C42 medium carbon steel wire rod after hot dip galvanizing (the weight of zinc on the wire rod surface was 550 g/m^2^). The hot-dip galvanized wire rod was drawn with the use of a multi-stage drawing machine in seven drafts using conventional and hydrodynamic dies with a drawing angle α = 5°, Table 1. The wire was drawn at a speed of 5 to 20 m/s. Calcium-sodium grease was used as a lubricant in the drawing process. Table 2 presents the designations of the variants of the wires used for the tests.

### 2.2. Methodology of Scanning Research

The study of the microstructure of the zinc coating was carried out with the use of the SEM scanning electron microscope. The initial material was tested—wire rod with a diameter of 5.5 mm and final wires with a diameter of 2.2 mm, drawn by conventional and hydrodynamic methods at the speed 5, 10, 15 and 20 m/s. In order to emphasize the influence of the drawing method on the structure of the coating, only the microstructures of wires with a diameter of 2.2 mm (wires after drawing in 7 drafts) are presented at two extreme drawing speeds, namely, 5 and 20 m/s. The paper analyzes the microstructure of the zinc coating on the longitudinal and transverse cross-sections to the drawing direction. Scanning tests were performed at various magnifications. For wires of such a diameter and such a coating, the positive effect of drawing in hydrodynamic dies on the zinc coating at a magnification of 2000× is most visible. Hence, the paper presents microstructures at such magnification.

### 2.3. Methodology of Corrosion Tests

Corrosion tests of conventionally and hydrodynamically drawn galvanized wires were conducted following the EN ISO 6988 standard in an atmosphere with sulfur dioxide with general moisture condensation as a reflection of conditions in an industrial environment. For this purpose, an Erichsen Hydrotherm chamber (chamber temperature was approximately 40 °C) was used. The wires were formed into springs before being put into the chamber. The total residence time of the samples in the chamber was 336 man-hours (7 cycles x 6 days, 8 h each). After each of the seven cycles, the surface of the zinc coatings was analyzed and the mass of the samples was measured. There was white corrosion on the surface of the samples.

The tests were supplemented by corrosion tests in sprayed brine carried out in a Kesternich chamber in accordance with the EN ISO 9227 standard (tests were conducted at 35 °C, the concentration of atomized sodium chloride in distilled water was 5%). Similarly, as in the case of corrosion tests in an atmosphere with sulfur dioxide, samples of springs made of the galvanized wire were used for corrosion tests in a salt chamber. After each of the cycles, the surface of the zinc coatings was analyzed, and the mass of the samples was measured (one cycle is 24 h of wire/spring in a salt spray chamber).

Springs made of 2.2 mm diameter wires were prepared for the test in the chamber (the diameter of the coils was 22 mm). There were three springs for each variant of the wire. All of the samples were cleaned, numbered and weighed before testing. In order to verify the loss of the mass of the original sample caused by corrosion, the wires were weighed after the last test cycle, and then chemically and mechanically cleaned as per ISO 8407 using an ultrasonic cleaner.

## 3. The Metallographic Analysis of the Zinc Coating

**Figure 1:**
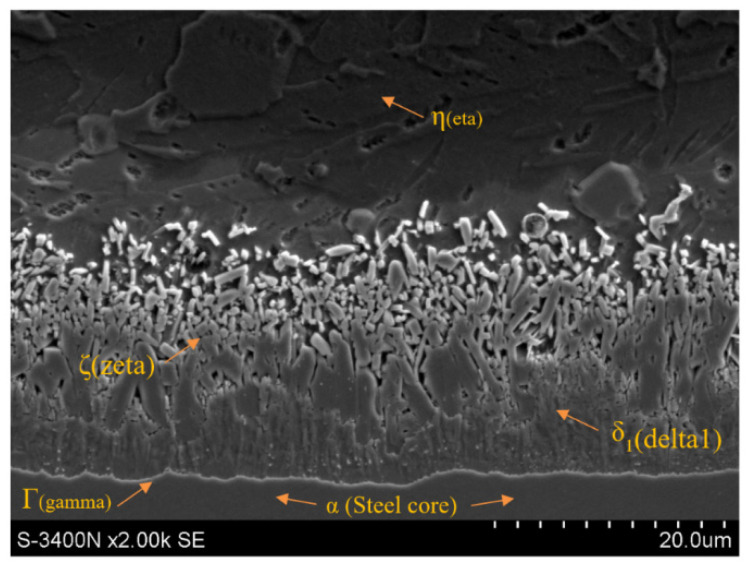
The microstructure of the zinc coating on the surface of the wire rod with the marks.

The first stage of the metallographic analysis was the analysis of the phase equilibrium system of Fe–Zn alloys, which was the basis for the presentation of individual structure components and determination of their occurrence in zinc coatings. Figure 1 shows the morphology of the zinc dip coating on the surface of C42 (0.4% C) medium carbon steel wire rod.

Figure 1 shows that the zinc coating consists of two main layers. The first layer, directly adjacent to the substrate, is referred to as the diffusion or alloying layer and is formed as a result of complex reactions between iron and liquid zinc. The diffusion layer consists of the Γ (gamma), δ_1_ (delta1) and ζ (zeta) phases. Directly on the steel substrate, the intermetallic phase Γ, containing 20.5–28% iron and marked with the formula Fe3Zn10, grows in a thin layer. Then, there is the phase δ_1_ containing 7–11.5% iron, determined by the formula FeZn7. In the areas of the coating richer in zinc, the intermetallic phase ζ containing 6 to 6.2% of iron, described by the formula FeZn13, increases and is located directly adjacent to the second outer (unalloyed) layer of the zinc coating containing pure zinc. This phase is denoted in the literature by the symbol η.

**Figure 2:**
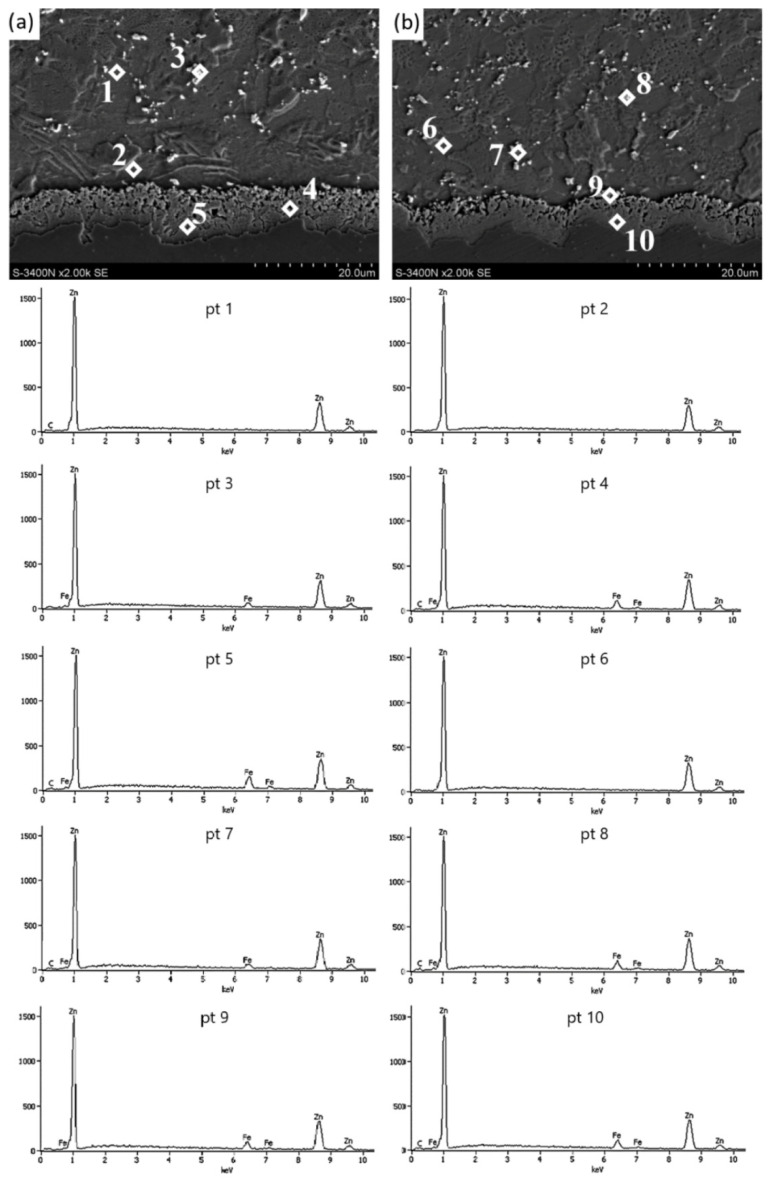
The (SEM) coating microstructure and the EDS X-ray spectral analysis in the micro-areas of the coating on the wire cross-section from variants (**a**) K5 and (**b**) H5.

The microstructure of the zinc coating shown in Figure 1 is a typical coating after hot-dip galvanizing in the unhardened state. In the drawing process, intensive plastic deformation of the coating occurs, it is accompanied by its heating and thinning as it passes through successive drawing dies. The dynamics of these phenomena depends on the adopted drawing technology, including the type of dies. Figure 2 and Table 3 present the results of tests for drawn wires in conventional and hydrodynamic dies with a speed of v = 5 m/s.

**Figure 3:**
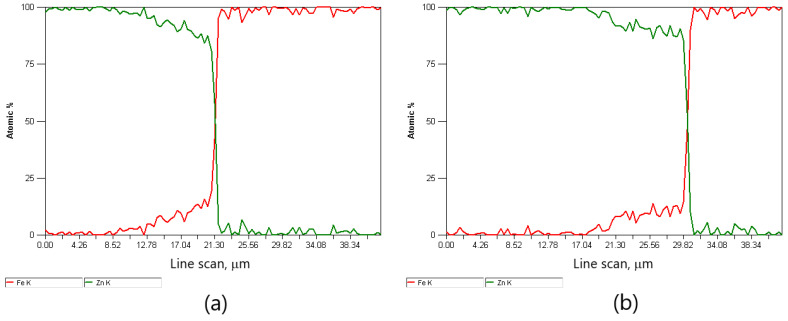
The EDS X-ray spectral analysis in coating micro-areas on the cross-section of the galvanized variant wire (**a**) K5 and (**b**) H5.

The microstructure of the zinc coating in the wires of the K5 and H5 variants shown in Figure 2 confirmed, irrespective of the drawing method, the presence of irregular intermetallic phase boundaries on the cross-section, which can be considered a normal phenomenon after the drawing process. Nevertheless, the greater irregularity of the boundary between the diffusion layer and the α-phase occurs in the K5 variant, while the course of this boundary in the H5 variant resembles a sinusoidal shape, which may be related to the favorable effect of the lubricant on the zinc coating in the airless die. The thick layer of grease allowed the soft zinc coating to deform more uniformly. Therefore, in the H5 variant, the repeatability of the shape of the interfacial boundary is visible. Better lubrication conditions during hydrodynamic drawing also contributed to obtaining a thicker zinc coating (Figure 3).

Tests on the amount of zinc on the surface of the wires showed that the wires drawn with the hydrodynamic method at a speed of 5 m/s showed the highest coating thickness. The microstructure of zinc coatings shown in Figure 2 divides it into an outer layer and a diffusion layer. The outer layer consists of pure zinc and intermetallic phase inclusions, which is confirmed by the analysis of the chemical composition of the micro-areas with cracked crystals at points 3, 7 and 8 (Figure 2), with the corresponding mass iron content of 4.8%, 4.7% and 6.8%, respectively.

In the conventional drawing, higher friction favors bidirectional diffusion and the development of the diffusion layer. This is confirmed by the chemical analysis of the micro-areas in points 4 and 5 with iron content, respectively, by masses of 7.1% and 10.0% corresponding to the phase’s δ_1_ stability (Figure 2a). In the H5 variant, in selected areas in the upper zone of the diffusion layer, there are areas with a lower iron content of 6.9% by mass (Point 9, Figure 2b), which correspond to phase ζ. However, it is difficult to distinguish the boundary of this phase in the coating structure, but its residual presence is noticeable in the hydrodynamic method.

**Figure 4:**
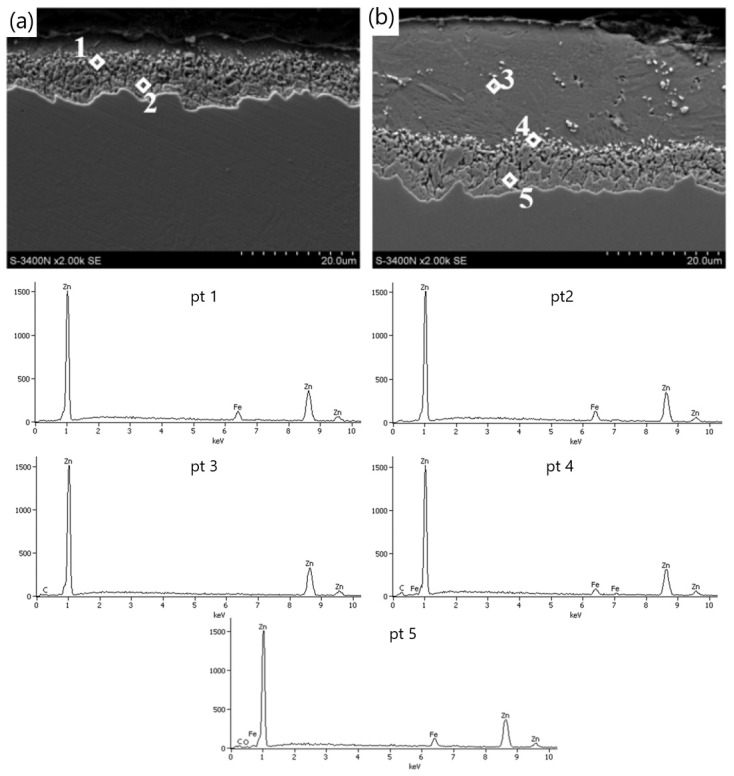
The (SEM) coating microstructure and EDS X-ray spectral analysis in the micro-areas of the coating on the cross-section of the wire from variants (**a**) K20 and (**b**) H20.

**Figure 5:**
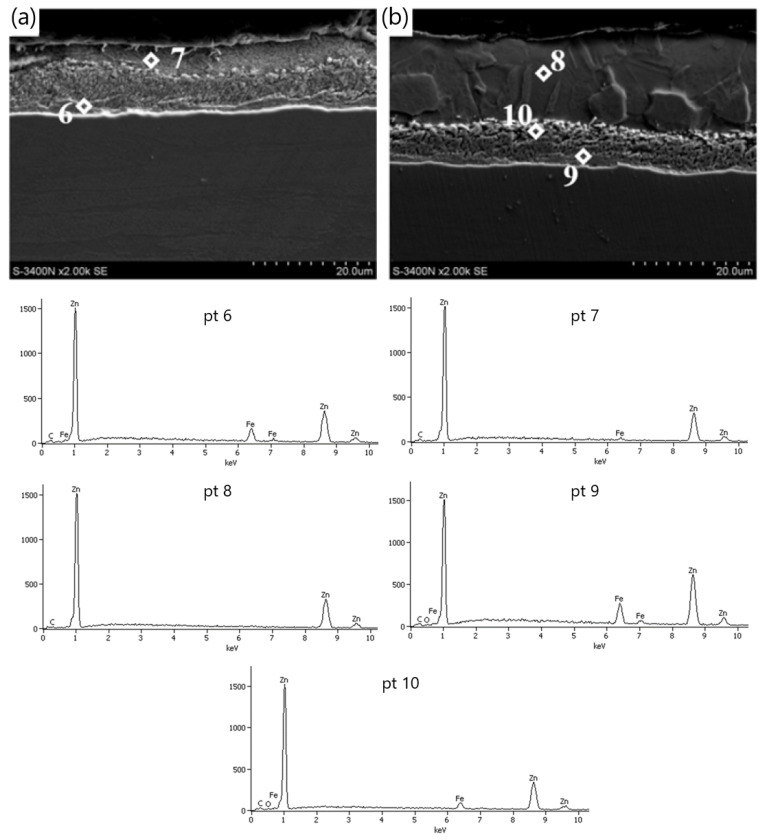
The (SEM) coating microstructure and EDS X-ray spectral analysis in the micro-areas of the coating on the longitudinal cross-section of the wire from variants (**a**) K20 and (**b**) H20.

The percentage loss of the cross-section due to plastic processing causes the cracking of crystals with low iron content. This results in dispersion in the outer layer made of pure zinc. The analysis of the variants allowed to state that at the drawing speed of 5 m/s, differences between the drawing methods can already be noticed. In the hydrodynamic method, there is a sinusoidal boundary between the diffusion layer and the phaseα, whereas, in the conventional method, the boundary formation trend cannot be assigned. The chemical analysis of the micro-areas shows that the ζ phase is residual in the H5 variant, but its boundary is not visible, which is a result of deformation caused by plastic processing. In the K5 variant, in the studied micro-areas, the stability was established only for phase δ_1_ in the upper and lower part of the diffusion layer. This could be due to higher temperatures due to greater friction. The effect of this is to increase the iron content in the diffusion layer, where the ζ phase has reacted, turning into phase δ_1_. The results presented have confirmed the influence of the drawing method on the microstructure of the zinc coating. It can be expected that the increase in the drawing speed from 5 to 20 m/s should contribute to the creation of even greater differences between the analyzed variants. This is confirmed by Figure 4 and Figure 5 and Table 4.

**Figure 6:**
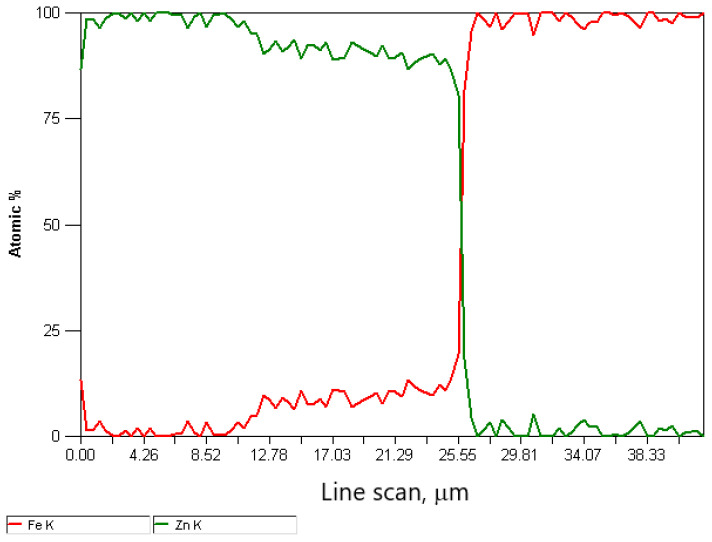
The EDS X-ray spectral analysis in the coating micro-areas on the cross-section of the galvanized H20 variant wire.

**Figure 7:**
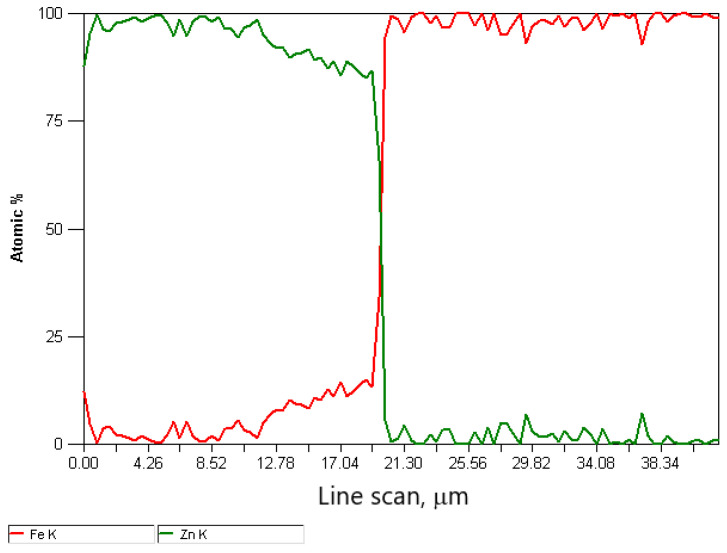
The EDS spectral X-ray analysis in the micro-areas of the coating on the cross-section of the galvanized K20 wire.

The difference in the thickness of the zinc coating of the variants is presented in Figure 6 and Figure 7. The in-depth analysis of the cross-section of the thickness of the zinc coating was expressed in microns depending on the percentage of intermetallic phases, marking the marked border from which the steel core of the wire begins. Thus, this analysis showed a significant influence of the drawing method on the thickness of the zinc coating on the wire after the high-speed multistage drawing. The visible green and red lines denoting zinc and iron, respectively, show the boundary between the zinc coating and the core at their intersection. The differences between the variants K20 (conventional method) and H20 (hydrodynamic method) are more than 5 μm. It can also be stated that the higher the drawing speed, the more visible the positive influence of hydrodynamic drawing on the thickness and formation of the zinc coating in the multistage drawing process of steel wires.

The decrease in the thickness of the zinc coating in the conventionally drawn wires at high speeds in relation to wires drawn analogously in hydrodynamic dies is confirmed by the microstructures of zinc coatings on the wire cross-section presented in Figure 4 and Figure 5. In the K20 variant (Figure 4a), a significant decrease in the thickness of the coating was noted, which translated into a decrease in the thickness of the unalloyed zinc phase. Hence, the outer layer is hardly visible. The thinning of the outer layer was simultaneously accompanied by a marked growth of the diffusion phase. The increased friction at the wire/drawing die interface in the conventional method resulted, on the one hand, in abrasion of the outer layer and, on the other hand, is an increase in the temperature and growth of the diffusion layer. The thin outer layer has no visible crystal cracks. It is supposed that with such a high temperature and a thin outer layer, they could have dissolved in pure zinc. The analysis of the chemical composition in the diffusion layer (Figure 4a) showed that in points 1 and 2, the iron content was, respectively, by mass 7.5% and 9.5%, which corresponds to δ_1_ phase stability. However, point 7, marked in Figure 5a, contains a mass of 1.6% of iron in the outer layer. This supports the theory that, when drawing at high speeds, the dissolution of low iron crystals in a thin outer layer composed of pure zinc occurs. The analysis of the microstructure with a longitudinal cross-section (Figure 5a) in point 6 with iron content by mass of 10.2% confirms the occurrence of phase δ_1_; however, determining its boundaries after the plastic processing is very difficult.

The metallographic analysis of the structures presented in Figure 6 confirmed the presence of a thicker zinc coating on the wires drawn with the hydrodynamic method at a speed of 20 m/s. The microstructure of the H20 variant, compared to the K20 variant, shows a relatively thick outer layer and a thinner diffusion layer. This means that the negative impact of friction on the material is partially reduced during the drawing process with the hydrodynamic method. This is shown by the research on the microstructure of the coatings (Figure 4b and Figure 5b). The outer layer of the H20 variant with a Zn content equal to 100% by mass (point 3 and point 8) visible in the cross-section and longitudinal section is thicker than that of the K20 variant and is characterized by the intermetallic inclusions. However, in the conventional method, these inclusions are dissolved at high drawing speeds, of 20 m/s, due to the high temperature.

The chemical analysis of the micro-areas in points 4 and 10 (Figure 4b and Figure 5b) showed that the iron content of the above-mentioned points is 6.0% and 6.4% by weight, respectively, which confirms the presence of a residual phase ζ. Under the Fe–Zn phase equilibrium system, the ζ phase is not stable at temperatures exceeding 520 °C [22]. This proves that the wire drawn with the hydrodynamic method is lower in temperature. The ζ phase boundary was deformed as a result of the plastic working, which leads to the lack of its differentiation, and a small amount is the result of its reconstruction into phase δ_1_. During high-speed plastic processing, this phase develops (Figure 4b and Figure 5b), which is confirmed by the micro-areas in points 5 and 9 containing 8.0% and 10.8% by weight, respectively, which corresponds to the stability of phase δ_1_.

The analysis carried out on the microstructure of the zinc coating for different drawing methods confirms that the drawing speed and the method affect the microstructure of the zinc coating. The greater influence of friction in the conventional method contributes to the decrease in the thickness of the surface layer and the increase in the diffusion layer in relation to the hydrodynamic method, the higher the drawing speed, the greater the differences between the analyzed drawing methods. In the conventional method, when drawing at high speeds, v = 20 m/s, intense heating of the top layer of the wire occurs, which leads to bidirectional diffusion and complete transformation of the ζ in phase δ_1_. In the hydrodynamic method, at a speed of 20 m/s, in the analyzed micro-areas, some places indicate the presence of the ζ phase, partially dispersed in the layer with pure zinc.

## 4. Corrosion Tests

**Figure 8:**
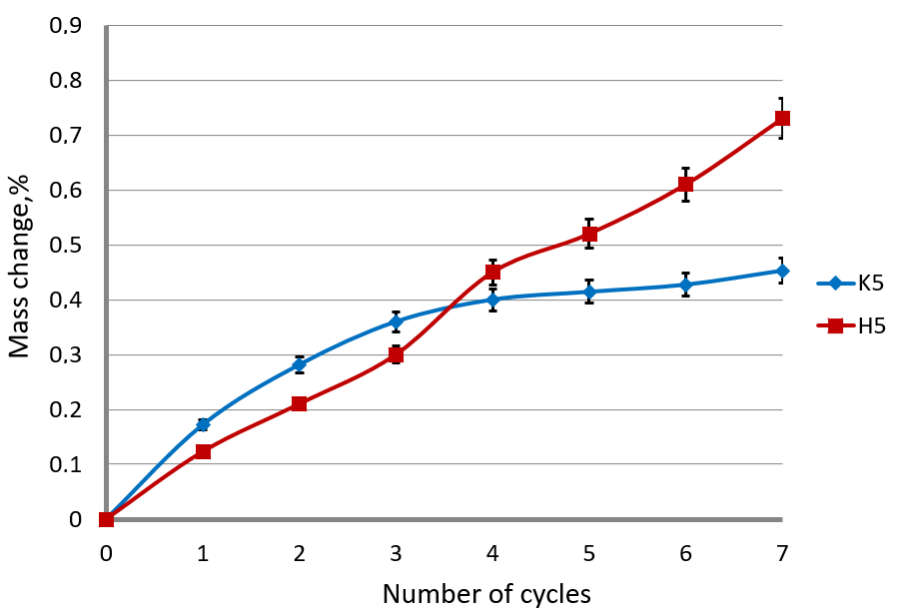
The mass change of the galvanized wires drawn with the conventional (K5) and hydrodynamic (H5) method with a speed of 5 m/s as a function of the number of exposure cycles in the sulfur dioxide chamber.

**Figure 9:**
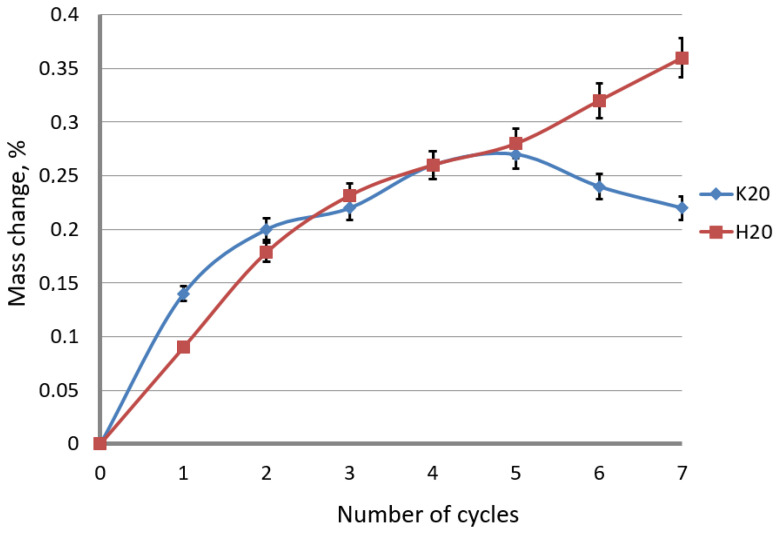
The mass change of the galvanized wires drawn with the conventional (K20) and hydrodynamic (H20) method with the speed of 20 m/s as a function of the number of exposure cycles in the sulfur dioxide chamber.

Corrosion tests carried out in the study confirmed the influence of the drawing method on the corrosion resistance of hot-dip galvanized steel wires. The results of the tests are shown in Figure 8 and Figure 9.

The conducted tests confirmed the significant influence of the drawing method on the corrosion resistance of the wires in the sulfur dioxide chamber. In the conventional method, a rapid increase in the mass of the samples due to corrosion products is initially visible, while after the third cycle, there is a regress to the last test series. Dynamic growth at the initial stage of research affects the speed of development in subsequent cycles. The inhibition of the corrosion process is believed to be in the thinner phase η in the K5 surface layer in relation to the H5 variant. In the variant drawn with the hydrodynamic method, uniform unit weight of the samples is noticeable. A thicker surface layer of pure zinc is able to produce more corrosion products during the entire test. This process lengthens the corrosion course of the successive intermetallic phases. Hence, it can be concluded that hydrodynamically drawn wires have higher corrosion resistance than conventional drawn wires.

The influence of the drawing method on the corrosion process of the galvanized wire caused by the environment of sulfur dioxide should be considered in terms of the mechanism of corrosion formation on zinc coatings. At low drawing speeds, of the order of 5 m/s, in the conventional method, a non-linear increase in mass was observed, while for the hydrodynamic method, the mass change approximately increased proportionally to the number of cycles. The same tendency of weight increase was noted for the high drawing speeds (variant H20). The conventional wire drawing at a speed of 20 m/s, on the other hand, causes an increase in the mass of samples supplemented with corrosion products, and then after cycle 5, it decreases. The weight loss was caused by a loss of corrosion products that had been formed during the tests.

The coating encased with the corrosion products, which took place in the first stage, slows down the development of corrosion until the products detach; it was the case in the K20 variant after the 5th cycle. It creates free areas for free corrosion development. It should also be taken into account that the conventionally drawn wires have a more extensive diffusion layer, which slows down the corrosion process of the intermetallic phases, which contributed to an increase in the corrosion resistance of this variant. The greater thickness of the zinc coating, and, in particular, of the η phase, in the method of the hydrodynamic drawing at a speed of 20 m/s, has an impact on higher corrosion resistance. In the first stage of the corrosion development, the key is the thickness of the pure zinc phase, which is the substrate of the corrosion products. The uniform increase in mass by the corrosion products, visible in Figure 9, in the hydrodynamic method, shows the continuous expansion of white corrosion, which is an insulator contributing to the increase in corrosion resistance in the hydrodynamic method. In the corrosion test with sulfur dioxide, where only the layer of pure zinc was subject to corrosion, it is impossible to objectively determine the overall corrosion course in the selected variants. Different layer thicknesses ambiguously allow determining the corrosion resistance. It has been established from the research that a thicker surface layer allows for the expansion of the products, partially isolating the corrosion process, while at a drawing speed of 20 m/s in the conventional method, the drawing speed contributed to the development of the diffusion layer, inhibiting the development of red corrosion in the second stage.

**Figure 10:**
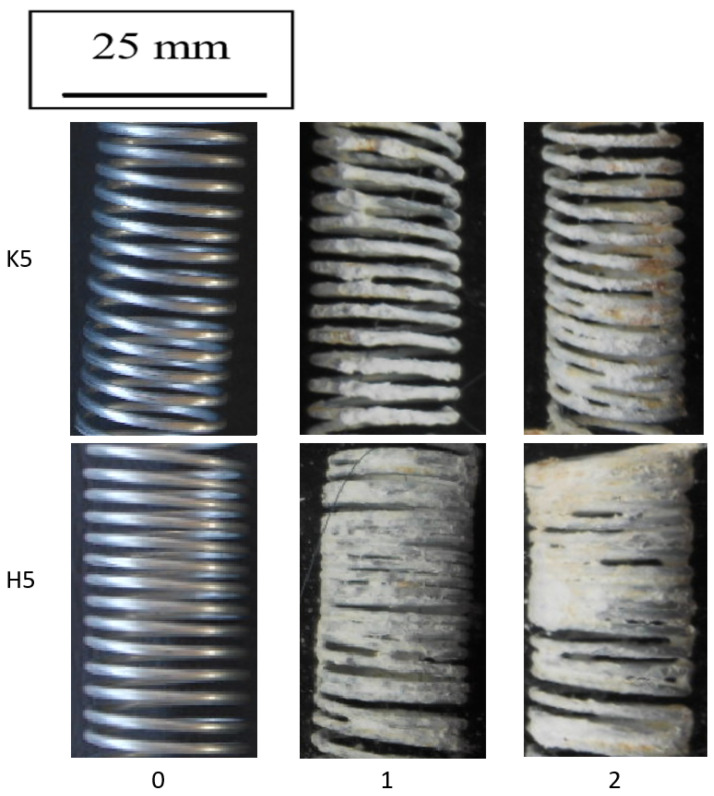
The surface appearance of the wires drawn at a speed of 5 m/s using the conventional K5 and hydrodynamic H5 method during corrosion tests in a salt chamber after two test cycles.

**Figure 11:**
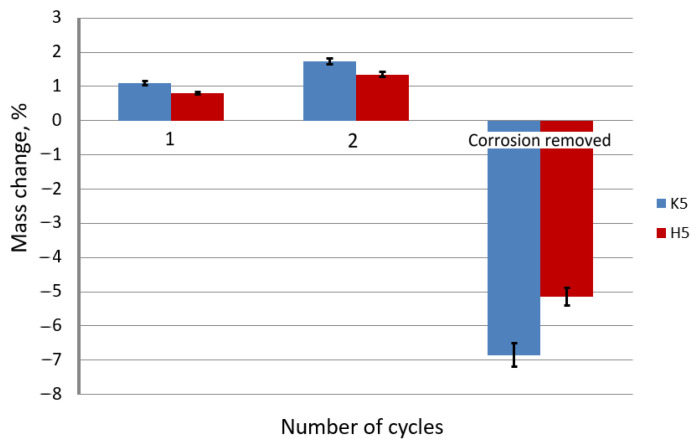
The percentage change in mass of the galvanized wire samples drawn at a speed of 5 m/s using the conventional K5 method and the hydrodynamic H5 method, made in a salt spray chamber, where the exposure took place in cycles.

The corrosion tests in sprayed brine confirmed that in more aggressive corrosive conditions the drawing method plays a key role in corrosion resistance. Figure 10 shows the photographic documentation after each cycle of the selectively selected samples of drawn wires at a speed of 5 m/s, while Figure 11 shows the unit weight change after each test cycle.

**Figure 12:**
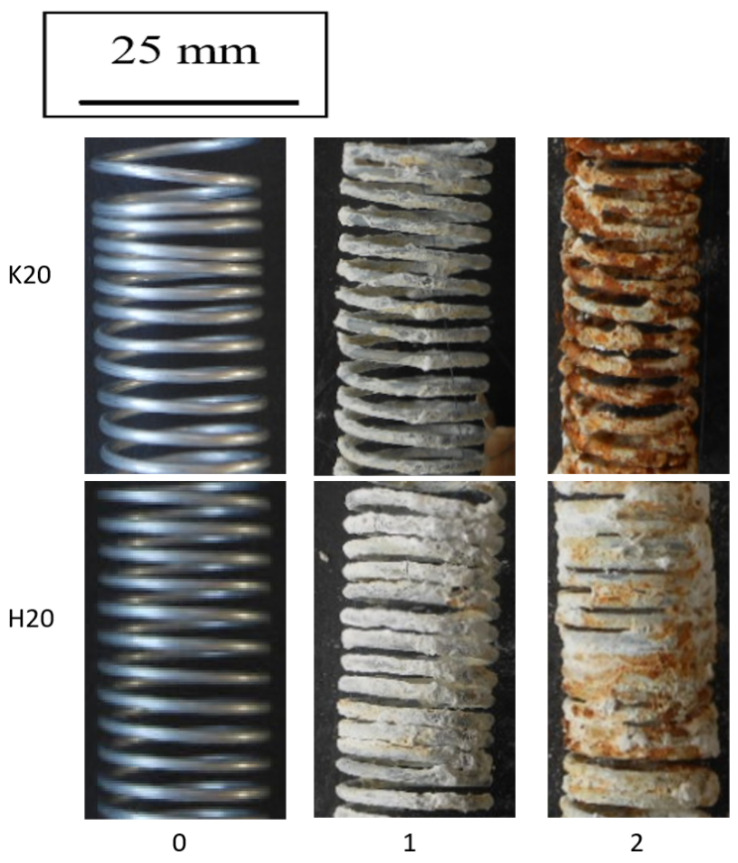
The surface appearance of the wires drawn at a speed of 20 m/s using the conventional K20 and hydrodynamic H20 method during corrosion tests in a salt chamber after two test cycles.

**Figure 13:**
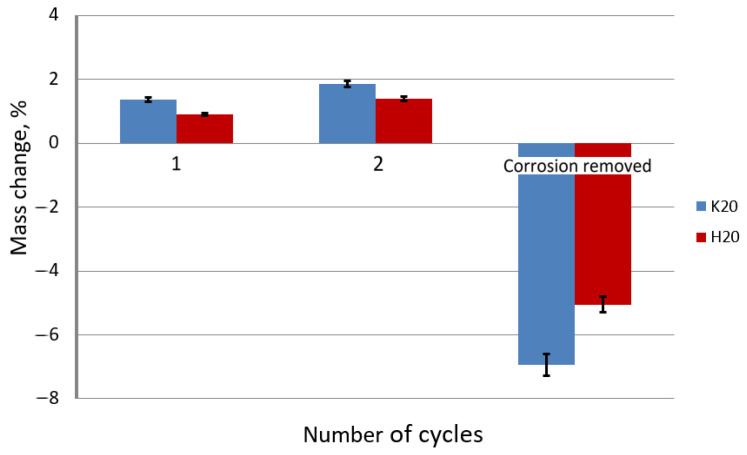
The percentage change in mass of the galvanized wire samples drawn at a speed of 20 m/s using the conventional K20 method and the hydrodynamic H20 method, made in a salt spray chamber, where the exposure took place in cycles.

On the basis of Figure 10 and Figure 11 it can be concluded that the drawing method significantly influences the speed of the corrosion process in the wires after the drawing process. The use of the hydrodynamic dies in the multistage drawing of the galvanized wires has a positive effect on the corrosion process. The greater mass of the pure zinc phase on the drawn wires in the hydrodynamic method causes the production of a greater amount of corrosion products, which insulates and slows down the process. In addition, it should be taken into account that in the hydrodynamic method, the lower residual stresses than in the conventional method (confirmed in earlier work [40]) do not contribute to the detachment of the insulating layer of white corrosion, which is shown in Figure 11. The results obtained from the percentage change in the mass of the samples confirmed the greater mass of the samples after two test cycles in the hydrodynamic method, but after cleaning, the greater weight loss was recorded for the conventional method. Such a situation takes place when low residual stresses do not affect the detachment of the white corrosion layer in the hydrodynamic method, unlike the conventional method, where new areas are created in the place of the detached products, accelerating the corrosion process, which in turn creates a greater deficit in mass. The positive effect of hydrodynamic drawing on the corrosion resistance was also confirmed by tests on drawn wires at a speed of 20 m/s (Figure 12 and Figure 13).

Based on the data presented in Figure 12 and Figure 13, it can be concluded that the drawing method has a significant impact on the corrosion process. The galvanized wires drawn at a speed of 20 m/s showed a lower corrosion resistance than those drawn at a speed of 5 m/s. Corrosion tests conducted in a salt spray chamber on the samples drawn with a drag speed of 20 m/s showed the mechanism of the corrosion process, taking into account the differences between the conventional and hydrodynamic methods. As in the case of drawing at a speed of 5 m/s, a more significant percentage increase in the weight of the wires drawn with the conventional method is visible compared to the hydrodynamic method, which is due to the same corrosion development mechanism. The greater mass in the surface layer of pure zinc, which was the substrate of the corrosion product, reacted to form insulation from the white corrosion produced during the first test cycle. The white corrosion produced during the second test cycle created such a tight coating that only discoloration indicative of preliminary corrosion of the intermetallic phases is visible. However, when referring to the conventional method, in the second test cycle, red corrosion products are visible on the entire sample, indicating a greater loss of corrosion resistance (Figure 12). The greater percentage of weight loss shown in Figure 13 confirms the lower corrosion resistance of the conventional method in relation to the hydrodynamic method, both at the speed of 5 m/s and 20 m/s.

The greater corrosion resistance of hydrodynamically drawn wires is also related to the positive effect of the lubricant on the zinc coating. There is a large increase in lubricant pressure during the multistage drawing. The literature [39] shows that the pressure of the lubricant in the airless die, at speed exceeding 20 m/s, may exceed 2000 MPa. Hence, according to the author, the compressive stresses generated in this process on the circumference of the wire could close microcracks on the wire surface, which additionally sealed the zinc coating.

The analysis of the results shows that the corrosion process of the galvanized wires depends on the technology of wire drawing. The drawing technology, including the speed and method of drawing, affect not only the distribution of individual phases in the zinc coating, but above all its thickness. Along with the increase in total draft, gradual thinning of the zinc coating occurs. This is due to the elongation of the material and grinding of the coating in the dies. Increasing the drawing speed increases the temperature of the wire surface, which causes the coating to soften and stripping it faster. On the other hand, the use of hydrodynamic strings allows to reduce the friction at the interface between the wire and the die, which lowers the temperature of the wire. As a consequence, hydrodynamically drawn wires are characterized by a thicker zinc coating, which determines the corrosion resistance. The performed tests have shown that the higher the drawing speed, the more dynamic the corrosion development. However, the use of the hydrodynamic method significantly reduces the corrosion rate of galvanized steel wires.

## 5. Conclusions

This increase in friction at the wire/die interface in the conventional method, compared to the hydrodynamic method, contributed to the decrease in the thickness of the coating and the increase in the diffusion layer. With the increase in the drawing speed, the differences between the analyzed drawing methods also increase. In the conventional method, while drawing at high speeds v = 20 m/s, there was a two-way diffusion and complete remodeling of the ζ phase in δ_1_. In the hydrodynamic method, at the speed of 20 m/s, in the analyzed micro-areas, places showing the presence of the ζ phase, partially dispersed in the layer with pure zinc, were observed. Thus, the drawing method has a significant effect on the structure and thickness of the zinc coating. The use of the hydrodynamic dies in the multistage drawing process allows for producing a wire with a thicker zinc coating, characterized by a less developed diffusion layer. As an input for the metal products, this material is characterized by high corrosion resistance, and the zinc coating on its surface has better plastic deformation properties. It has been shown that, regardless of the drawing method, an increase in the drawing speed of the galvanized wires causes a decrease in their corrosion resistance, especially in the conventionally drawn wires.

## Figures and Tables

**Table 1:** Distribution of single reductions, G_p_, the total reduction, G_c_ and drawing speed v.

Draw No.	0	1	2	3	4	5	6	7
φ, mm	5.50	4.73	4.10	3.57	3.13	2.77	2.46	2.20
G_p_, %	-	26.04	24.86	24.18	23.13	21.68	21.13	20.02
G_c_, %	-	26.04	44.43	57.87	67.61	74.64	79.99	84.00
v, m/s	-	1.06	1.43	1.90	2.47	3.15	4.00	5
		2.12	2.86	3.80	4.94	6.31	8.00	10
		3.17	4.28	5.70	7.41	9.46	12.00	15
		4.22	5.70	7.59	9.88	12.62	16.00	20

**Table 2:** Marking of technological variants of wires with zinc coatings, where v is the drawing speed and δ is the the zinc mass on the φ 2.2 mm wire surface.

Drawing Method	Marking	v, m/s	Variant	δ, g/m^2^
Conventional	K	5	K5	215
		10	K10	176
		15	K15	159
		20	K20	127
Hydrodynamic	H	5	H5	223
		10	H10	202
		15	H15	204
		20	H20	186

**Table 3:** Chemical composition in selected micro-areas of the coating obtained on the wire in variants K5 and H5.

Point of Measure	Content of Elements			
	Fe-K		Zn-K	
	%Mass	%at.	%Mass	%at.
point 1	-	-	100.0	100.0
point 2	-	-	100.0	100.0
point 3	4.8	5.6	95.2	94.4
point 4	7.1	8,2	92.9	91.8
point 5	10.0	11.5	90.0	88.5
point 6	-	-	100.0	100.0
point 7	4.7	5.4	95.3	94.6
point 8	6.8	7.9	93.2	92.1
point 9	6.9	8.0	93.1	92.0
point 10	7.6	8.8	92.4	91.2

**Table 4:** Chemical composition of the selected micro-areas of the coating obtained on the wire in the K20 and H20 variant.

Point of Measure	Content of Elements			
	Fe-K		Zn-K	
	%Mass	%at.	%Mass	%at.
point 1	7.5	8.7	92.5	91.3
point 2	9.2	10.6	90.8	89.4
point 3	-	-	100.0	100.0
point 4	6.0	6.9	94.0	93.1
point 5	8.0	9.2	92.0	90.8
point 6	10.2	11.8	89.8	88.2
point 7	1.6	1.9	98.4	98.1
point 8	-	-	100.0	100.0
point 9	10.8	12.4	89.2	87.6
point 10	6.4	7.4	93.6	92.6

## Data Availability

Not applicable.
